# A multi-scene deep learning model for automated segmentation of acute vertebral compression fractures from radiographs: a multicenter cohort study

**DOI:** 10.1186/s13244-024-01861-y

**Published:** 2024-12-02

**Authors:** Hao Zhang, Genji Yuan, Ziyue Zhang, Xiang Guo, Ruixiang Xu, Tongshuai Xu, Xin Zhong, Meng Kong, Kai Zhu, Xuexiao Ma

**Affiliations:** 1 Department of Spinal Surgery, The Affiliated Hospital of Qingdao University, Qingdao, China; 2 College of Computer Science and Technology, Qingdao University, Qingdao, China; 3 Department of Ophthalmology, Qingdao Central Hospital, University of Health and Rehabilitation Sciences (Qingdao Central Hospital), Qingdao, China; 4 Department of Spinal Surgery, The Affiliated Hospital of Weifang Medical University, Weifang, China; 5 Department of Pain, YanTai YuHuangDing Hospital, Yantai, China; 6 Department of Spinal Surgery, Binzhou Medical University Hospital, Binzhou, China; 7 Department of Radiology, The Affiliated Hospital of Qingdao University, Qingdao, China; 8 Department of Spinal Surgery, Qingdao Municipal Hospital, Qingdao, China

**Keywords:** Deep learning, Fractures, Compression, Spine, Radiography

## Abstract

**Objective:**

To develop a multi-scene model that can automatically segment acute vertebral compression fractures (VCFs) from spine radiographs.

**Methods:**

In this multicenter study, we collected radiographs from five hospitals (Hospitals A–E) between November 2016 and October 2019. The study included participants with acute VCFs, as well as healthy controls. For the development of the Positioning and Focus Network (PFNet), we used a training dataset consisting of 1071 participants from Hospitals A and B. The validation dataset included 458 participants from Hospitals A and B, whereas external test datasets 1–3 included 301 participants from Hospital C, 223 from Hospital D, and 261 from Hospital E, respectively. We evaluated the segmentation performance of the PFNet model and compared it with previously described approaches. Additionally, we used qualitative comparison and gradient-weighted class activation mapping (Grad-CAM) to explain the feature learning and segmentation results of the PFNet model.

**Results:**

The PFNet model achieved accuracies of 99.93%, 98.53%, 99.21%, and 100% for the segmentation of acute VCFs in the validation dataset and external test datasets 1–3, respectively. The receiver operating characteristic curves comparing the four models across the validation and external test datasets consistently showed that the PFNet model outperformed other approaches, achieving the highest values for all measures. The qualitative comparison and Grad-CAM provided an intuitive view of the interpretability and effectiveness of our PFNet model.

**Conclusion:**

In this study, we successfully developed a multi-scene model based on spine radiographs for precise preoperative and intraoperative segmentation of acute VCFs.

**Critical relevance statement:**

Our PFNet model demonstrated high accuracy in multi-scene segmentation in clinical settings, making it a significant advancement in this field.

**Key Points:**

- This study developed the first multi-scene deep learning model capable of segmenting acute VCFs from spine radiographs.
- The model’s architecture consists of two crucial modules: an attention-guided module and a supervised decoding module.
- The exceptional generalization and consistently superior performance of our model were validated using multicenter external test datasets.

**Graphical Abstract:**

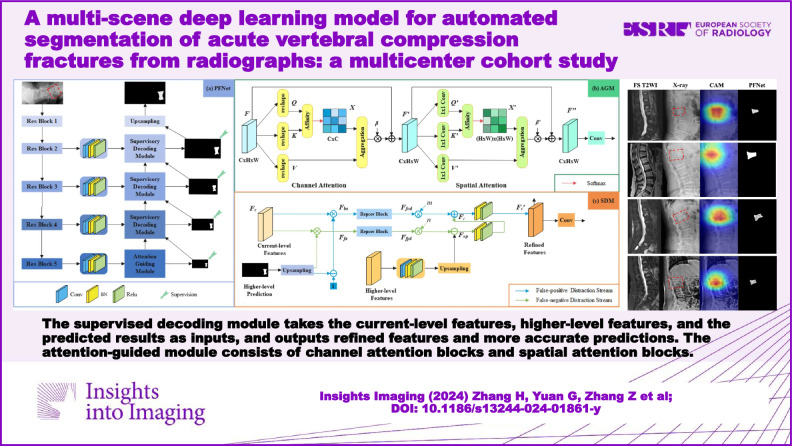

## Introduction

Vertebral compression fractures (VCFs), the most common type of spinal fracture, precipitate a cascade of adverse outcomes. These outcomes include back pain, reduced mobility, diminished quality of life, increased mortality rates, and the development of spinal kyphosis [1, 2]. Given these potential complications, timely and accurate diagnosis is essential.

Magnetic resonance imaging (MRI), with its exceptional contrast, is the gold-standard imaging modality used widely in clinical practice for the detection and diagnosis of acute VCFs. Acute VCFs exhibit distinct hyperintensity on T2-weighted imaging (T2WI) and fat-suppression T2WI sequences, differentiating them from normal vertebral bodies or chronic fractures. However, the similarity in MRI features between acute VCFs, Schmorl’s nodes, and type 1 Modic changes necessitates the development of new methods to effectively distinguish these conditions and avoid diagnostic errors [3–6].

Despite its utility, the complexity and time-consuming nature of MRI limits its accessibility, often delaying the diagnosis and subsequent treatment of acute VCFs. This delay can lead to prolonged patient discomfort and worsen the associated consequences [7]. Thus, ensuring prompt and accurate diagnosis is imperative for the timely treatment of patients with suspected acute VCFs.

While commonly used in clinical practice for its speed and efficiency, radiography has limitations in visualizing detailed anatomical structures necessary for identifying acute VCFs, often leading to diagnostic challenges [8, 9].

Recent advances in deep learning (DL), particularly in computer vision, have led to significant improvements in the processing of medical images. These advancements stem from the formidable feature learning capability of DL methodologies, which have played a crucial role in the classification and segmentation of radiographic images [10–14]. However, there remains a gap between the success of these models in research and their practical application in clinical settings, primarily due to challenges such as limited interpretability and restricted external generalizability.

The aim of this study is to introduce a novel DL model designed to identify, localize, and segment acute VCFs in both preoperative and intraoperative radiographs, offering a comprehensive solution that addresses the varied scenarios encountered in clinical practice. We seek to demonstrate that our model performs with high accuracy and provides superior interpretability and results when compared to existing DL segmentation models, as evidenced by both internal and external validations.

## Methods

### Study design and datasets

This multicenter study was conducted in accordance with the ethical principles of the Declaration of Helsinki and approved by the appropriate Ethics Committees. Informed consent was waived as this retrospective study utilized preexisting medical data. The work adhered to the Strengthening the Reporting of Cohort Studies in Surgery (STROCSS) criteria [15].

For this study, we collected spine radiographs from five hospitals across China to develop and validate our model. Our study cohort consisted of participants who underwent spinal MRI examinations between November 2016 and October 2019 and were diagnosed with acute VCFs in these five hospitals. To ensure the accuracy and precision of the model and to eliminate interference from healthy spinal conditions or chronic VCFs, we also included healthy participants. These MRIs showed no signs of acute VCFs and were collected from the same five hospitals. Detailed inclusion and exclusion criteria, as well as specific hospital information, can be found in the Supplementary Appendix.

A total of 2314 participants were enrolled in this study. Among them, 1529 participants from two hospitals (A and B) were divided into training (*n* = 1071) and validation (*n* = 458) datasets based on patient identification numbers, with the first 70% allocated to the training dataset and the remaining 30% to the validation dataset. Although sample size calculations were not performed, external test datasets were prepared. We also obtained participants and images from Hospitals C (external test dataset 1, *n* = 301), D (external test dataset 2, *n* = 223), and E (external test dataset 3, *n* = 261), respectively. The three external test datasets were used to evaluate the external generalizability of our model.

### Ground truth labeling and segmentation

Spine radiographs were labeled and segmented based on corresponding spinal MRI findings. Two experienced spinal surgeons independently reviewed both MRI scans and radiographs (reader 1, X.G. and reader 2, M.K., with a minimum of 5 years of clinical experience each). Consensus-based manual segmentations were obtained through collaboration with a third expert (reader 3, X.Z., with 8 years of experience in interpreting MRI). Acute VCF segmentation involves delineating the contours of the fractured vertebral body to ensure precise segmentation of the entire lesion. Segmentation was not performed for cases involving healthy spines or chronic VCFs in this study.

### Radiological examination

Routine anteroposterior and lateral spine radiography was performed using various machines, including Digital Diagnost (Philip), and DRX-Evolution Plus (Carestream), Yiso (SIEMENS) systems. All MRI scans were acquired using 1.5-T or 3.0-T MRI instruments with multichannel phased-array spine coils. The image acquisition parameters and details of the radiography instruments at the five hospitals are presented in the Supplementary Appendix.

### Model architecture

**Fig. 1:**
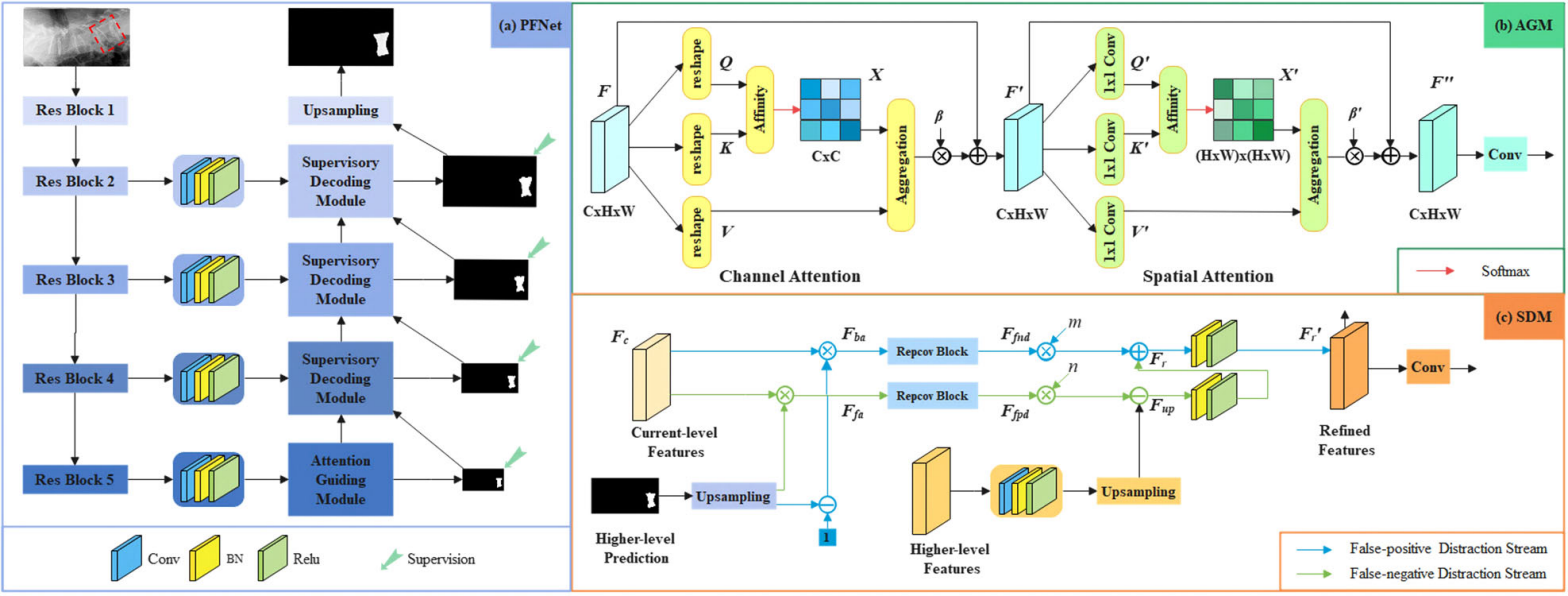
The architecture of (**a**) our PFNet model, (**b**) AGM, and (**c**) SDM

Our developed model, the Positioning and Focus Network (PFNet), was specifically designed for the identification and segmentation of acute VCFs in spine radiographs. The PFNet consists of two main modules: the attention-guided module (AGM) and the supervised decoding module (SDM) (Fig. 1). The AGM functions similarly to the clinical image interpretation process, conducting a global search across the entire radiograph to identify potential abnormalities associated with acute VCFs. In contrast, the SDM operates in the recognition phase, focusing on the target region for precise segmentation of acute VCFs. Its primary objective is to achieve accurate localization and detailed characterization of these fractures.

The workflow of the PFNet is as follows: first, a spine radiograph is input into the ResNet-50 [16] backbone network to extract multi-level features. These features then undergo channel reduction through four convolutional layers. Next, the AGM is applied to the deepest layer features to locate acute VCFs. Finally, multiple SDMs are used to progressively detect and eliminate false-positive and false-negative interferences in order to achieve accurate segmentation of acute VCFs (Fig. 1).

### Multi-scene test and model evaluation

We developed the model in the training dataset and independently tested it with the validation and external test datasets. The three distinct external test cohorts were specifically used to evaluate the external generalizability of the PFNet model. Additionally, 62 intraoperative radiographs were obtained from Hospital A to assess the segmentation performance of the PFNet model within the C-arm fluoroscopy dataset.

We compared our PFNet model to previous competitive segmentation approaches, including adjacent context coordination network (ACCoNet) [17], boundary-aware segmentation network (BASNet) [18], and Deepcrack (Deep) models [19], to verify the effectiveness of our PFNet model. Receiver operating characteristic (ROC) curves were used to evaluate the models’ segmentation of acute VCFs. Key metrics such as the area under the curve (AUC), accuracy, sensitivity, and specificity were calculated to compare performance across datasets and models. The object and background pixels were considered positive and negative classes, respectively. In addition, gradient-weighted class activation mapping (Grad-CAM) was applied to explain the feature learning and segmentation results of the PFNet model [20–22].

### Statistical analysis

All statistical analyses were performed using IBM SPSS Statistics for Windows, version 22.0. (IBM Corp., Armonk, NY, USA) and Python version 3.7. Normally distributed continuous variables were represented as means ± standard deviations, while categorical variables were presented as percentages (%). Student’s *t*-test and Mann–Whitney *U*-tests were used to compare continuous clinical variables, as appropriate. Chi-square or Fisher’s exact tests were used to compare categorical variables. Statistical significance was set at *p* < 0.05. The software liberties and packages used included pytorch (version 3.7.1), numpy (version 1.21.5), pandas (version 1.3.5), and torchvision (version 0.10.0).

## Results

### Population characteristics

Tables 1 and 2 lists the comprehensive characteristics of the participants in the training dataset, validation dataset, and external test datasets 1–3. In the study cohort of 2314 participants (mean age: 62.86 ± 13.76 years), 916 (39.59%) were men and 1398 (60.41%) were women. A total of 840 (36.30%) participants were diagnosed with acute VCFs.

**Table 1:** Baseline dataset characteristics

	All participants, (*n* = 2314)	Training dataset, (*n* = 1071)	Validation dataset, (*n* = 458)	Test dataset 1, (*n* = 301)	Test dataset 2, (*n* = 223)	Test dataset 3, (*n* = 261)
Age (years)	62.86 ± 13.76	63.39 ± 13.12	63.82 ± 12.83	64.11 ± 12.96	63.04 ± 15.55	57.42 ± 15.84
Sex, no. (%)						
Male	916 (39.59%)	405 (37.82%)	181 (39.52%)	120 (39.86%)	90 (40.36%)	120 (45.98%)
Female	1398 (60.41%)	666 (62.18%)	277 (60.48%)	181 (60.13%)	133 (59.64%)	141 (54.02%)
Acute VCFs cases, no. (%)	840 (36.30%)	406 (37.91%)	174 (38.00%)	94 (31.23%)	102 (45.74%)	64 (24.52%)
Acute VCFs distribution, no. (%)						
Thoracic	403 (17.42%)	206 (19.23%)	137 (29.91%)	32 (10.63%)	4 (1.79%)	24 (9.20%)
Lumbar	437 (18.89%)	200 (18.67%)	37 (8.08%)	62 (20.60%)	98 (43.95%)	40 (15.33%)

**Table 2:** Detail characteristics of acute VCFs in each dataset

	Training dataset, (*n* = 1071)			Validation dataset, (*n* = 458)			External test dataset 1, (*n* = 301)			External test dataset 2, (*n* = 223)			External test dataset 3, (*n* = 261)		
	Acute VCFs, (*n* = 406)	non-Acute VCFs, (*n* = 665)	*p*	Acute VCFs, (*n* = 174)	non-Acute VCFs, (*n* = 284)	*p*	Acute VCFs, (*n* = 94)	non-Acute VCFs, (*n* = 207)	*p*	Acute VCFs, (*n* = 102)	non-Acute VCFs, (*n* = 121)	*p*	Acute VCFs, (*n* = 64)	non-Acute VCFs, (*n* = 197)	*p*
Age (years)	71.26 ± 11.36	58.53 ± 11.81	< 0.001	71.21 ± 10.67	59.29 ± 11.91	< 0.001	72.34 ± 9.77	60.37 ± 12.51	< 0.001	70.08 ± 12.58	57.12 ± 15.39	< 0.001	66.88 ± 10.92	54.35 ± 16.00	< 0.001
Sex, no. (%)			< 0.001			< 0.001			0.031			0.012			< 0.001
Male	106 (26.11%)	299 (44.96%)		44 (25.29%)	137 (48.24%)		29 (30.85%)	91 (43.96%)		32 (31.37%)	58 (47.93%)		11 (17.19%)	109 (55.33%)	
Female	300 (73.89%)	366 (55.04%)		130 (74.71%)	147 (51.76%)		65 (69.15%)	116 (56.04%)		70 (68.63%)	63 (52.07)		53 (82.81%)	88 (44.67%)	
Acute VCFs distribution, no. (%)															
Thoracic	206 (50.74%)	–		137 (78.74%)	–		32 (34.04%)	–		4 (3.92%)	–		24 (37.50%)	–	
Lumbar	200 (49.26%)	–		37 (21.26%)	–		62 (65.96%)	–		98 (96.08%)	–		40 (62.50%)	–	

The training dataset consisted of 1071 participants (mean age: 63.39 ± 13.12 years, 405 men and 666 women). Among them, 406 individuals (37.91%) were diagnosed with acute VCFs, including 106 men (26.11%) and 300 women (73.89%). The validation dataset consisted of 458 participants, with a mean age of 63.82 ± 12.83 years. Among them were 174 cases of acute VCFs (38.00%), with 137 (29.91%) involving thoracic VCFs and 37 (8.08%) involving lumbar VCFs. The proportions of participants diagnosed with acute VCFs varied across the external test datasets: 31.23%, 45.74%, and 24.52% for external test datasets 1–3, respectively.

### Segmentation performance of the PFNet

**Fig. 2:**
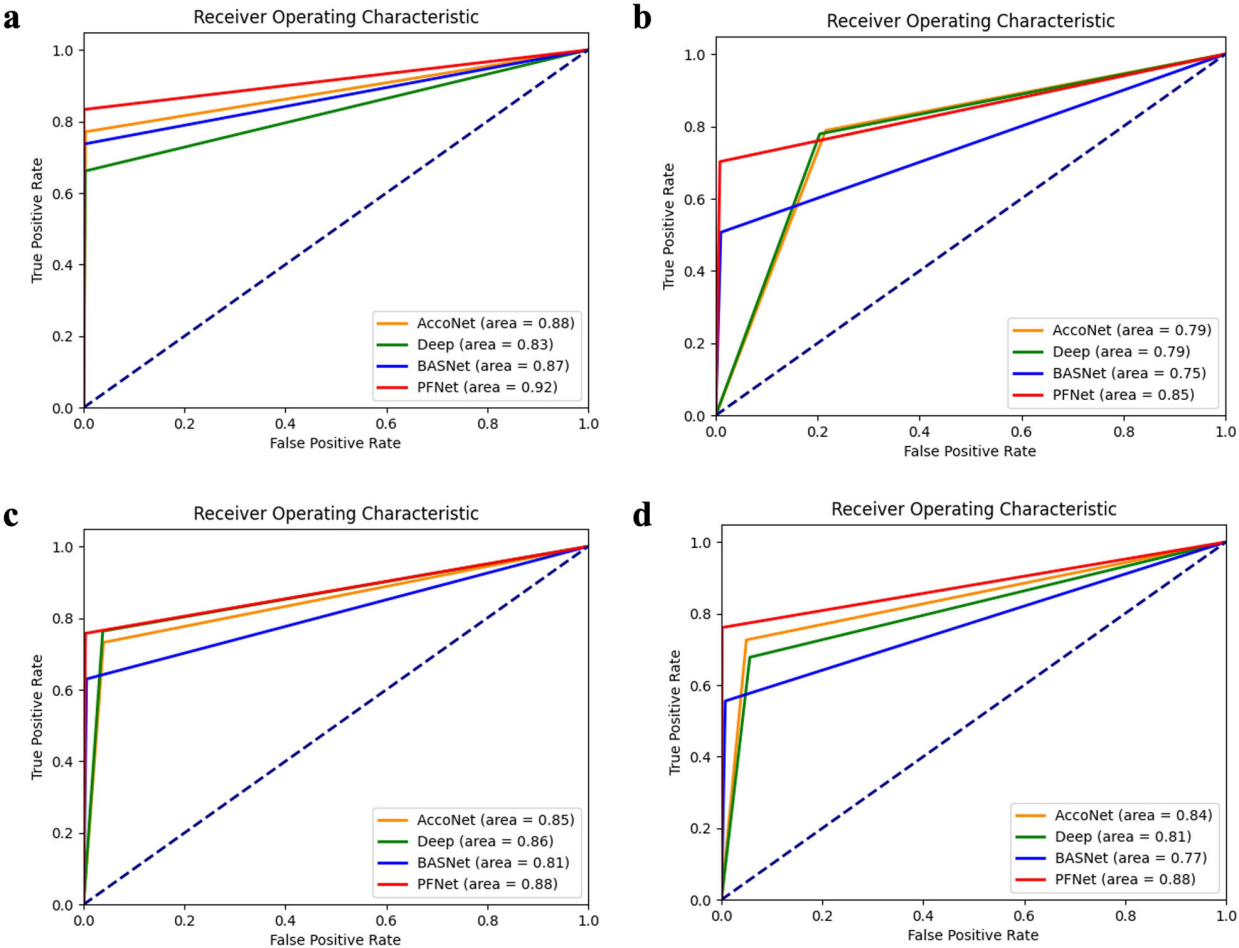
Performance of the models. ROC curves of the PFNet, ACCoNet, BASNet, and Deep models in (**a**) the validation dataset, (**b**) external test dataset 1 (**c**) external test dataset 2, and (**d**) external test dataset 3

We compared the performance of our PFNet model with other competitive models using both the validation and external test datasets. Our findings consistently demonstrated that the PFNet outperformed or was on par with the other models across all metrics and datasets (Fig. 2 and Table 3). Particularly in the three external test datasets, the PFNet showed exceptional accuracy, sensitivity, and specificity values of 99.21%, 75.47%, and 99.59%, respectively. Moreover, the ROC curves comparing the four models across the validation and external test datasets consistently showed that the PFNet had the highest AUC values. For instance, the AUC values for the ACCoNet were 0.88, 0.79, 0.85, and 0.84 in the validation and external test datasets 1–3, respectively. In contrast, the AUC values for the PFNet were 0.92, 0.85, 0.88, and 0.88, respectively.

**Table 3:** Performance of the models in the validation and external test datasets

	PFNet	ACCoNet	BASNet	Deep
Validation dataset				
Accuracy (%)	99.93	99.9	99.81	99.72
Sensitivity (%)	99.94	99.92	99.83	99.76
Specificity (%)	98.63	97.04	96.08	94.86
External test dataset 1				
Accuracy (%)	98.53	79.62	97.75	80.28
Sensitivity (%)	75.62	80.78	53.58	80.08
Specificity (%)	99.24	79.58	99.13	80.29
External test dataset 2				
Accuracy (%)	99.21	95.78	98.9	95.96
Sensitivity (%)	75.47	75.5	63.79	78.42
Specificity (%)	99.59	96.1	99.46	96.24
External test dataset 3				
Accuracy (%)	100	95.07	98.78	94.34
Sensitivity (%)	78.61	76.54	58.43	71.53
Specificity (%)	100	95.3	99.29	94.62

**Fig. 3:**
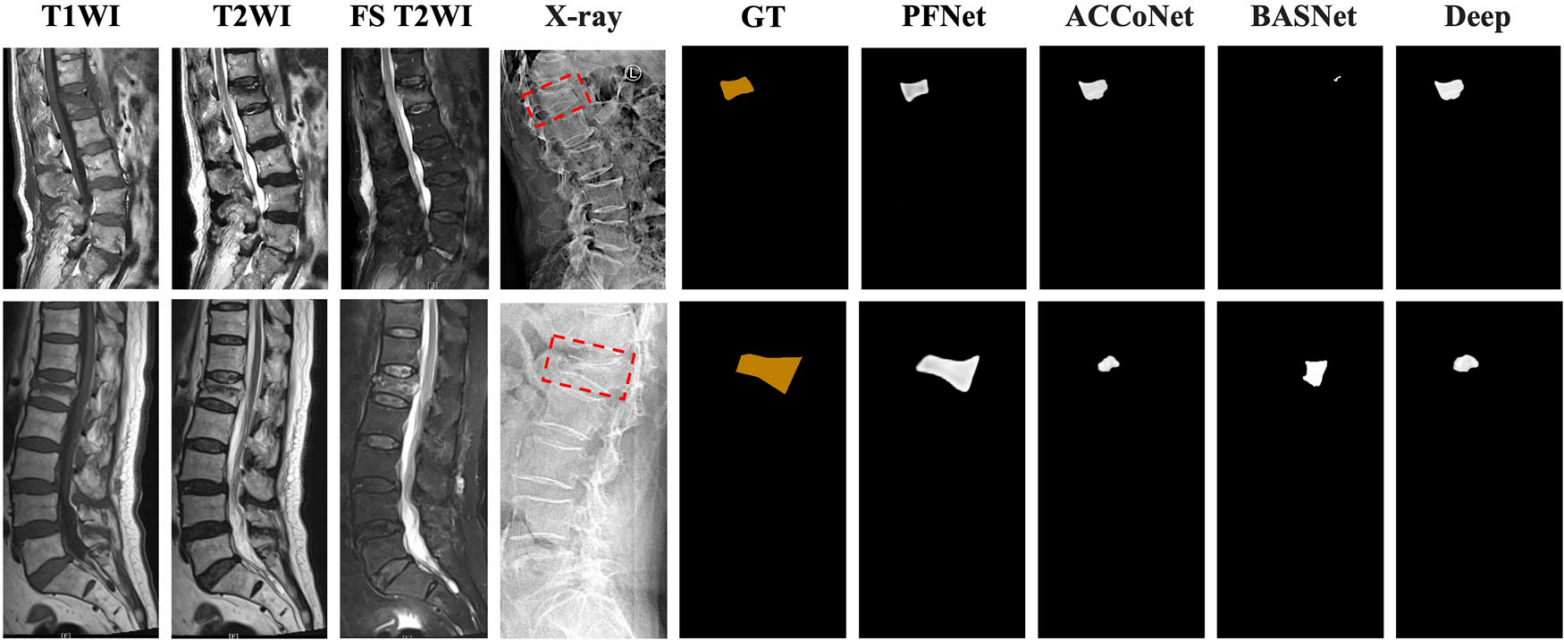
Comparison of the models’ segmentation results with MRI and ground truth (GT) data for acute VCFs. The images are derived from external test datasets, with acute VCFs highlighted by red boxes in the radiography image

**Fig. 4:**
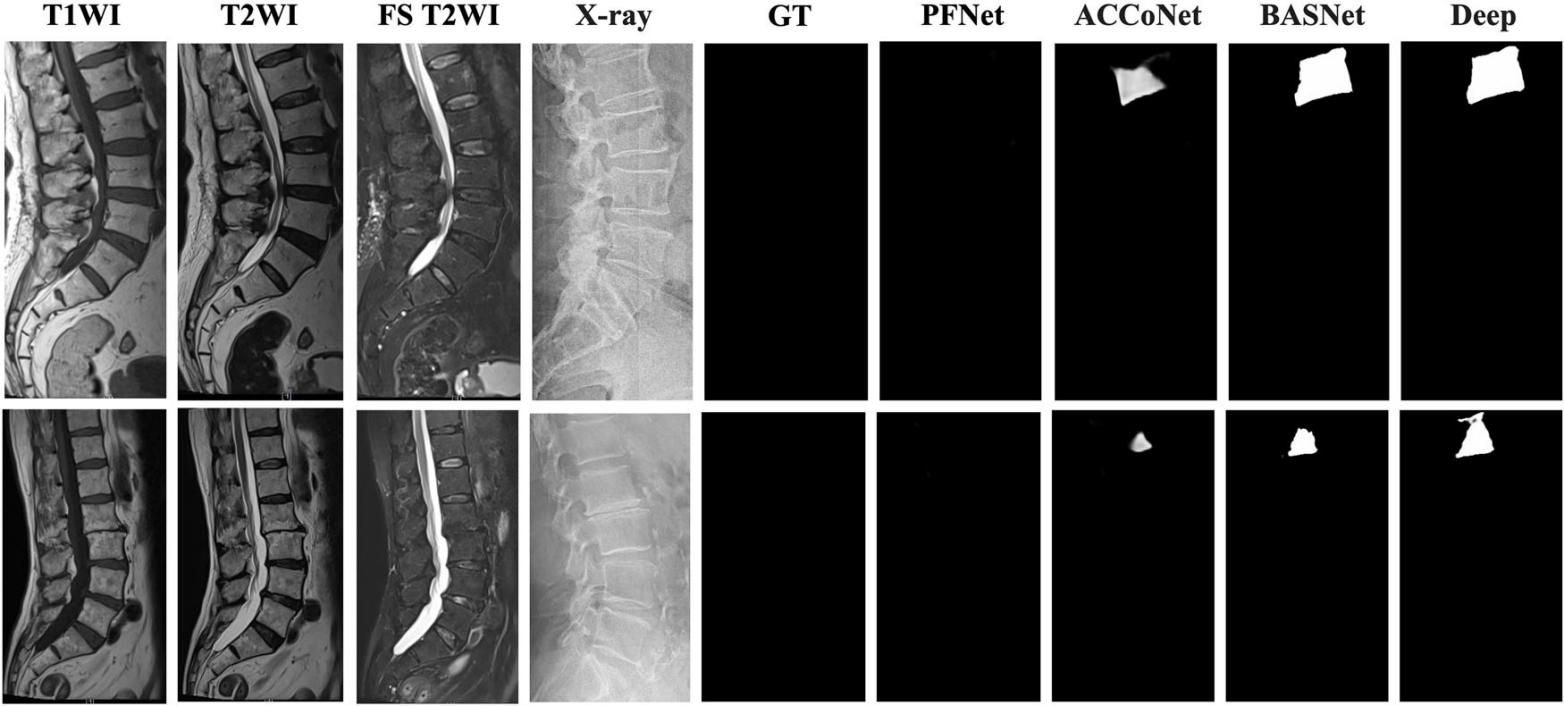
Comparison of the models’ segmentation results with MRI and ground truth (GT) data for normal spines (non-acute VCFs). The images are derived from external test datasets

We compared the PFNet to other models qualitatively. The PFNet consistently maintained coherence in the region and contour delineation of acute VCFs, showing its ability to understand semantic relationships between regions and contours through the SDM. This reduced boundary errors and improved segmentation performance (Fig. 3). Additionally, the PFNet showed segmentation accuracy comparable to MRI findings and ground truth in normal spinal radiographs or those with chronic VCFs. In contrast, other models had areas of erroneous segmentation due to limitations in decoding mechanisms (Fig. 4). This visual comparison clearly highlighted the PFNet’s superiority in segmentation accuracy and precision.

**Fig. 5:**
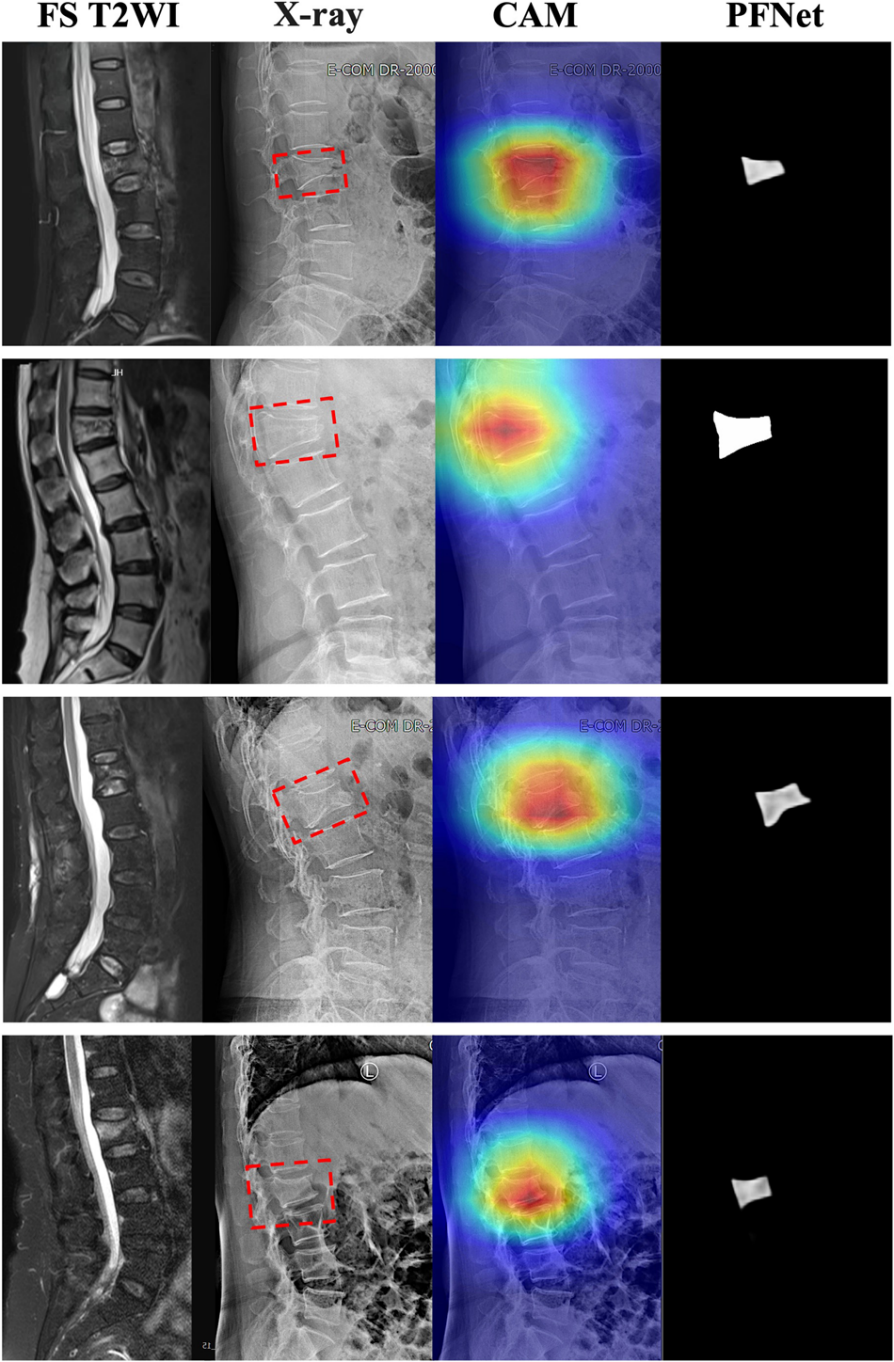
Attention heatmaps of the PFNet for representative participants in the external test datasets. The acute VCFs are highlighted by red boxes in the radiography image

The Grad-CAM technique (Fig. 5) provided an intuitive visualization of the feature attention maps in the PFNet, demonstrating the interpretability and effectiveness of our model. The PFNet accurately responded to and focused on the regions of acute VCFs, showcasing the efficacy of the attention mechanism. This visualization not only improved interpretability but also emphasized the model’s ability to identify and prioritize relevant features, contributing to its exceptional performance in identifying and delineating acute VCFs.

### Multi-scene test

**Fig. 6:**
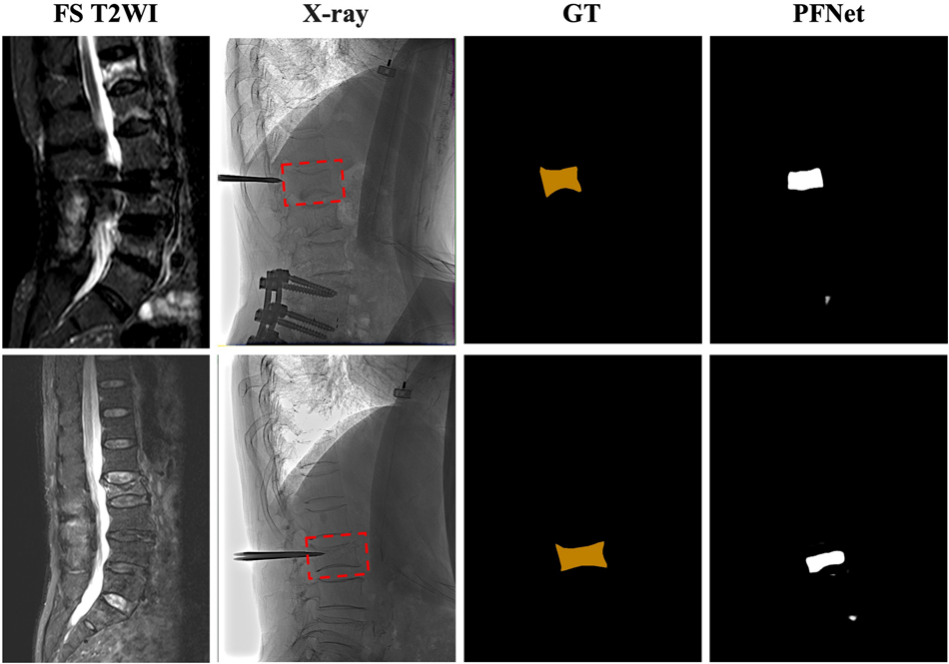
Segmentation results of the PFNet for representative participants in the C-arm fluoroscopy dataset. The acute VCFs are highlighted by red boxes in the radiography image

The performance and application of the PFNet model in intraoperative radiographs were evaluated using the C-arm fluoroscopy dataset. Within this dataset, the PFNet demonstrated accuracy and specificity rates of 98.56% and 99.36%, respectively. The Supplementary Appendix provides detailed demographics and the ROC curve of the PFNet in this dataset. While the PFNet is the first segmentation DL model applied to intraoperative radiographs, it did not achieve high sensitivity in the C-arm fluoroscopy dataset. However, the segmentation results were consistent within most regions of acute vertebral VCFs, despite potential limitations in accurately delineating the boundaries of acute VCFs. This consistency meets the demands and requirements for intraoperative localization, as shown in Fig. 6.

## Discussion

Clinicians must promptly and accurately distinguish acute VCFs from normal vertebrae, and chronic VCFs to facilitate early evaluation of the surgical strategy and determine the etiology of the acute VCFs. Although modern MRI techniques have successfully addressed the challenges of locating and identifying acute VCFs, patients often endure lengthy delays in undergoing MRI, resulting in pain and discomfort during the waiting period. The results of this study describe the development of a novel multi-scene DL algorithm specifically designed to segment acute VCFs within spine radiographs. We further confirmed the ability of this innovation through meticulous internal and external validation using a comprehensive multi-institutional dataset. As a multi-scene segmentation model, we rigorously evaluated the PFNet in both preoperative and intraoperative radiographs, demonstrating its performance and effectiveness in distinct clinical scenarios. Our comparative analysis against competitive models unequivocally demonstrated the consistent superiority of the PFNet, with unparalleled performance across all datasets and metrics. These findings confirmed that the PFNet is a leading model that excelled in accurately segmenting acute VCFs across diverse imaging scenarios.

### Advantages of the PFNet architecture

The high degree of similarity between acute VCFs and normal vertebral bodies in radiographs [14] can lead to false positive and false negative predictions in the initial segmentation results. To address these erroneous predictions, we designed an SDM, which takes current-level features, higher-level features, and the predicted results as inputs and outputs refined features and more accurate predictions. This module aims to refine the segmentation results by leveraging contextual information from multiple levels and incorporating feedback from higher-level features, ultimately improving the segmentation accuracy. Additionally, the AGM consists of channel attention blocks and spatial attention blocks. Both blocks employ non-local operations to capture long-range dependencies between channels and spatial positions, thereby enhancing the semantic representation of the deepest layer features from a global perspective.

### Robustness and multi-scenes

Unlike other DL models that have limited image availability for training and external validation [5, 14], our PFNet was developed using an extensive dataset obtained from two hospitals. Furthermore, we validated the robustness and reliability of the PFNet using three distinct and independent datasets from three separate hospitals. This comprehensive dataset and thorough validation support the credibility and generalizability of the PFNet, distinguishing it from models that are limited by data availability and validation. Additionally, the limited external generalizability of DL models significantly hinders their translation into clinical practice [7]. The radiographs used in this study were generated by various instruments, ensuring the reproducibility of the PFNet model’s excellent performance [23, 24]. These findings highlight the significant improvements made by the PFNet, demonstrating its superior performance not only within the validation dataset but also across multiple external test datasets, indicating its exceptional generalization and consistently superior performance.

To the best of our knowledge, the PFNet model is the first DL model applied to segmenting acute VCFs from intraoperative radiographs. Surgeons rely solely on bone markers in radiographs to identify acute VCFs intraoperatively. Consequently, the condition may be confused with diseased vertebral bodies, resulting in serious medical consequences. Our analysis of the C-arm fluoroscopy dataset demonstrated the efficacy of the PFNet as a multi-scene segmentation DL model in analyzing intraoperative radiographs. However, the sensitivity of the PFNet model did not meet expectations, unlike its success in other performance metrics. This performance discrepancy between preoperative and intraoperative scenarios can be attributed to several factors. Firstly, the limited availability of intraoperative training data and the resulting domain shift could significantly impact the model’s performance. Additionally, insufficient training data specific to intraoperative tasks likely contributed to its suboptimal performance. Moreover, various surgical factors and instruments, such as tissue manipulation and the presence of metal implants, can complicate image interpretation. These factors could have influenced the model’s performance in interpreting intraoperative radiographs, thus impacting its sensitivity. In conclusion, the limited availability of intraoperative radiographs for training, coupled with insufficient data for the unique challenges posed by intraoperative scenarios and potential confounding surgical factors, collectively contribute to the suboptimal sensitivity observed in the performance of the PFNet within this context.

### Fully automated workflow streamlining

Accurately segmenting anatomical structures within medical images presents a crucial challenge for clinical diagnosis and analysis [25, 26]. In clinical settings, clinicians and radiologists dedicate significant time and effort to segmenting and delineating regions of interest within medical images. Therefore, the accurate and automated segmentation of medical images can alleviate the laborious and time-consuming burden on radiologists [27, 28].

The operational workflow of our PFNet model involves two main aspects: identifying acute VCFs and segmenting fractured vertebral bodies. Notably, previous studies have also developed DL models for segmenting anatomical structures in radiological images [29–32]. However, these models largely focused on normal or healthy anatomical structures, potentially falling short in effectively addressing clinical needs for disease diagnosis. Furthermore, previous studies have misunderstood the identification of acute VCFs as a simple classification task. This has led to models that lack interpretability and hinder their clinical usefulness. Previously, diagnosing and detecting acute VCFs in spinal radiographs required segmenting each vertebral body from the entire spine [5]. However, conventional models that combined classification and segmentation modules were often cumbersome and computationally complex. This could result in potential feature loss or overfitting. In contrast, our PFNet model excels in directly segmenting acute VCFs, thereby improving the clinical workflow and enhancing the efficiency of segmentation and diagnosis.

### Limitations

The PFNet model has some limitations. Firstly, although we have implemented the model in intraoperative radiographs, obtaining a substantial number of these images for training purposes remains challenging. Therefore, a larger and more diverse training set of intraoperative radiographs is needed to ensure the robustness and stability of the model. Secondly, owing to the retrospective study design, selection bias and inherent differences were inevitable. Therefore, prospective trials are needed and validation in prospective datasets is necessary to support the conclusions of this study. Additionally, we speculate that the bone density, fracture severity, degree of vertebral compression, and image quality may affect our model’s performance. As shown above, the PFNet model did not achieve high sensitivity in the C-arm fluoroscopy dataset. These issues may be attributed to factors such as insufficient data, low-quality images, and the complexity of specific patterns in C-arm fluoroscopy, among others.

To address these limitations, future work should focus on optimizing the algorithm through various means. Potential optimization tools include using image enhancement techniques (such as Retinex [33]) to preprocess the dataset to further improve segmentation accuracy. Additionally, incorporating mathematical models to constrain and guide the prediction results, such as the alternating direction method of multipliers algorithm, would provide a stronger mathematical basis for the model during prediction. Furthermore, continued testing and validation across diverse datasets will be essential to refine the algorithm’s accuracy and ensure its robustness in different practical applications. By addressing these limitations and exploring optimization avenues, the algorithm’s potential impact on decision-making processes can be fully realized, leading to more reliable and efficient outcomes in everyday practice.

## Conclusions

In this study, we developed a multi-scene DL model based on spine radiographs that enabled accurate preoperative and intraoperative segmentation of acute VCFs. Our PFNet model demonstrated excellent detection and segmentation performance. This model may serve as a valuable support tool for the diagnosis and localization of acute VCFs in clinical practice.

## Supplementary information

ELECTRONIC SUPPLEMENTARY MATERIAL

## Data Availability

The datasets used and/or analyzed during the current study are available from the corresponding author upon reasonable request.

## Abbreviations

ACCoNet: Adjacent context coordination network
AGM: Attention-guided module
AUC: Area under the curve
BASNet: Boundary-aware segmentation network
Deep: Deepcrack
DL: Deep learning
Grad-CAM: Gradient-weighted class activation mapping
MRI: Magnetic resonance imaging
PFNet: Positioning and focus network
ROC: Receiver operating characteristic
SDM: Supervised decoding module
T2WI: T2-weighted imaging
VCF: Vertebral compression fracture

## References

[1] Goldstein CL, Chutkan NB, Choma TJ, Orr RD (2015) Management of the elderly with vertebral compression fractures. Neurosurgery 77:S33–S45 26378356 10.1227/NEU.0000000000000947

[2] Petritsch B, Kosmala A, Weng AM et al (2017) Vertebral compression fractures: third-generation dual-energy CT for detection of bone marrow edema at visual and quantitative analyses. Radiology 284:161–168 28240561 10.1148/radiol.2017162165

[3] Modic MT, Steinberg PM, Ross JS, Masaryk TJ, Carter JR (1988) Degenerative disk disease: assessment of changes in vertebral body marrow with MR imaging. Radiology 166:193–199 3336678 10.1148/radiology.166.1.3336678

[4] Kaup M, Wichmann JL, Scholtz JE et al (2016) Dual-energy CT-based display of bone marrow edema in osteoporotic vertebral compression fractures: impact on diagnostic accuracy of radiologists with varying levels of experience in correlation to MR imaging. Radiology 280:510–519 26928067 10.1148/radiol.2016150472

[5] Xu F, Xiong Y, Ye G et al (2023) Deep learning-based artificial intelligence model for classification of vertebral compression fractures: a multicenter diagnostic study. Front Endocrinol 14:1025749 10.3389/fendo.2023.1025749 PMC10073698 37033240

[6] Liu J, Hao L, Zhang X et al (2018) Painful Schmorl’s nodes treated by discography and discoblock. Eur Spine J 27:13–18 28194524 10.1007/s00586-017-4996-8

[7] Zhang H, Yuan G, Wang C et al (2023) Differentiation of benign versus malignant indistinguishable vertebral compression fractures by different machine learning with MRI-based radiomic features. Eur Radiol 33:5069–5076 37099176 10.1007/s00330-023-09678-x

[8] Kim KC, Cho HC, Jang TJ, Choi JM, Seo JK (2021) Automatic detection and segmentation of lumbar vertebrae from X-ray images for compression fracture evaluation. Comput Methods Programs Biomed 200:105833 33250283 10.1016/j.cmpb.2020.105833

[9] Musbahi O, Ali AM, Hassany H, Mobasheri R (2018) Vertebral compression fractures. Br J Hosp Med 79:36–40 10.12968/hmed.2018.79.1.36 29315051

[10] He Y, Pan I, Bao B et al (2020) Deep learning-based classification of primary bone tumors on radiographs: a preliminary study. EBioMedicine 62:103121 33232868 10.1016/j.ebiom.2020.103121 PMC7689511

[11] Gao Y, Zeng S, Xu X et al (2022) Deep learning-enabled pelvic ultrasound images for accurate diagnosis of ovarian cancer in China: a retrospective, multicentre, diagnostic study. Lancet Digit Health 4:e179–e187 35216752 10.1016/S2589-7500(21)00278-8

[12] Jiang Y, Zhang Z, Yuan Q et al (2022) Predicting peritoneal recurrence and disease-free survival from CT images in gastric cancer with multitask deep learning: a retrospective study. Lancet Digit Health 4:e340–e350 35461691 10.1016/S2589-7500(22)00040-1

[13] Ueda D, Matsumoto T, Ehara S et al (2023) Artificial intelligence-based model to classify cardiac functions from chest radiographs: a multi-institutional, retrospective model development and validation study. Lancet Digit Health 5:e525–e533 37422342 10.1016/S2589-7500(23)00107-3

[14] Chen W, Liu X, Li K et al (2022) A deep-learning model for identifying fresh vertebral compression fractures on digital radiography. Eur Radiol 32:1496–1505 34553256 10.1007/s00330-021-08247-4

[15] Mathew G, Agha R, Albrecht J et al (2021) STROCSS 2021: strengthening the reporting of cohort, cross-sectional and case-control studies in surgery. Int J Surg 96:106165 34774726 10.1016/j.ijsu.2021.106165

[16] Lin CL, Wu KC (2023) Development of revised ResNet-50 for diabetic retinopathy detection. BMC Bioinform 24:157 10.1186/s12859-023-05293-1 PMC10114328 37076790

[17] Li G, Liu Z, Zeng D, Lin W, Ling H (2023) Adjacent context coordination network for salient object detection in optical remote sensing images. IEEE Trans Cybern 53:526–538 35417367 10.1109/TCYB.2022.3162945

[18] Wang R, Chen S, Ji C, Fan J, Li Y (2022) Boundary-aware context neural network for medical image segmentation. Med Image Anal 78:102395 35231851 10.1016/j.media.2022.102395

[19] Zou Q, Zhang Z, Li Q, Qi X, Wang Q, Wang S (2018) DeepCrack: learning hierarchical convolutional features for crack detection. IEEE Trans Image Process 10.1109/TIP.2018.2878966 30387731

[20] Panwar H, Gupta PK, Siddiqui MK, Morales-Menendez R, Bhardwaj P, Singh V (2020) A deep learning and grad-CAM based color visualization approach for fast detection of COVID-19 cases using chest X-ray and CT-scan images. Chaos Solitons Fractals 140:110190 32836918 10.1016/j.chaos.2020.110190 PMC7413068

[21] Mu W, Jiang L, Shi Y et al (2021) Non-invasive measurement of PD-L1 status and prediction of immunotherapy response using deep learning of PET/CT images. J Immunother Cancer 9:e002118 34135101 10.1136/jitc-2020-002118 PMC8211060

[22] Liu C, Xie H, Zhang Y (2021) Self-supervised attention mechanism for pediatric bone age assessment with efficient weak annotation. IEEE Trans Med Imaging 40:2685–2697 33351757 10.1109/TMI.2020.3046672

[23] Wennmann M, Thierjung H, Bauer F et al (2022) Repeatability and reproducibility of ADC measurements and MRI signal intensity measurements of bone marrow in monoclonal plasma cell disorders: a prospective bi-institutional multiscanner, multiprotocol study. Invest Radiol 57:272–281 34839306 10.1097/RLI.0000000000000838

[24] Wennmann M, Bauer F, Klein A et al (2023) In vivo repeatability and multiscanner reproducibility of MRI radiomics features in patients with monoclonal plasma cell disorders: a prospective bi-institutional study. Invest Radiol 58:253–264 36165988 10.1097/RLI.0000000000000927

[25] Sahiner B, Pezeshk A, Hadjiiski LM et al (2019) Deep learning in medical imaging and radiation therapy. Med Phys 46:e1–e36 30367497 10.1002/mp.13264 PMC9560030

[26] Wang K, Zhang X, Lu Y, Zhang X, Zhang W (2022) CGRNet: contour-guided graph reasoning network for ambiguous biomedical image segmentation. Biomed Signal Process Control 75:103621

[27] Mo Y, Han C, Liu Y et al (2023) HoVer-trans: anatomy-aware hover-transformer for ROI-free breast cancer diagnosis in ultrasound images. IEEE Trans Med Imaging 42:1696–1706 37018705 10.1109/TMI.2023.3236011

[28] Kojima S, Kitaguchi D, Igaki T et al (2023) Deep-learning-based semantic segmentation of autonomic nerves from laparoscopic images of colorectal surgery: an experimental pilot study. Int J Surg 109:813–820 36999784 10.1097/JS9.0000000000000317 PMC10389575

[29] Huang J, Shen H, Wu J et al (2020) Spine explorer: a deep learning based fully automated program for efficient and reliable quantifications of the vertebrae and discs on sagittal lumbar spine MR images. Spine J 20:590–599 31759132 10.1016/j.spinee.2019.11.010

[30] Zheng HD, Sun YL, Kong DW et al (2022) Deep learning-based high-accuracy quantitation for lumbar intervertebral disc degeneration from MRI. Nat Commun 13:841 35149684 10.1038/s41467-022-28387-5 PMC8837609

[31] Al Arif S, Knapp K, Slabaugh G (2018) Fully automatic cervical vertebrae segmentation framework for X-ray images. Comput Methods Programs Biomed 157:95–111 29477438 10.1016/j.cmpb.2018.01.006

[32] Zhao S, Wu X, Chen B, Li S (2021) Automatic vertebrae recognition from arbitrary spine MRI images by a category-consistent self-calibration detection framework. Med Image Anal 67:101826 33075638 10.1016/j.media.2020.101826

[33] Wu T, Wu W, Yang Y, Fan FL, Zeng T (2023) Retinex image enhancement based on sequential decomposition with a plug-and-play framework. IEEE Trans Neural Netw Learn Syst 35:14559–14572 10.1109/TNNLS.2023.3280037 37279121

