# Supplementary material for: A multi-scene deep learning model for automated segmentation of acute vertebral compression fractures from radiographs: a multicenter cohort study
Source: Insights Imaging. 2024 Dec 2;15:290. doi: 10.1186/s13244-024-01861-y (PMC11612108; doi:10.1186/s13244-024-01861-y)
Supplement: Supplementary file 1 — ELECTRONIC SUPPLEMENTARY MATERIAL [file 13244_2024_1861_MOESM1_ESM.pdf]

A multi-scene deep learning model for automated segmentation of acute vertebral compression fractures from radiographs: a multicenter cohort study

ELECTRONIC SUPPLEMENTARY MATERIAL

Table of Contents:

Section S1: Supplementary Methods

1) The detailed inclusion and exclusion criteria.

2) Image acquisition parameters and the details of X-ray instruments for each hospital.

3) Detailed information of development processes, parameter, software, packages of the deep learning models.

Section S2: Supplementary Tables

Supplementary Table 1: Detail baseline characteristics of all external test datasets.

Supplementary Table 2: Detail characteristics of acute VCFs in all external test datasets.

Section S3: Supplementary Figures

Supplementary Figure 1: ROC curves in C-arm fluoroscopy dataset for PFNet model.

Section S4: References for the Supplementary Material

Insights Imaging (2024) Zhang H, Yuan G, Zhang Z, et al.

Section S1: Supplementary Methods

1) The detailed inclusion and exclusion criteria

The inclusion criteria were as follows:

(1) participants within 2 weeks of back pain and underwent spinal MR and X-ray.

(2) normal participants; or participants with acute VCFs (trauma or osteoporosis).

The exclusion criteria were as follows:

(1) more than 1 week between X-ray and MRI examinations.

(2) participants without complete raw data and qualified X-ray/MRI images.

2) Image acquisition parameters and the details of DR and MRI instruments for each institutions

Hospital A: The Affiliated Hospital of Qingdao University; a Digital Diagnost (Philip) and a DRX-EVOLUTION PIUS (Carestream), the voltages of the lumbar anteroposterior and lateral films were 75-85 kV, and the currents were 25-75 Ams;

Hospital B: The Affiliated Hospital of Weifang Medical University; a Digital Diagnost (CARESTREAM DirectView System), the voltages of the lumbar anteroposterior and lateral films were 78-85 kV, and the currents were 200-220 Ams;

Hospital C: Binzhou Medical University Hospital; a Yiso (SIEMENS), the voltages of the lumbar anteroposterior and lateral films were 75-85 kV, and the currents were 35-50 Ams;

Hospital D: Qingdao Municipal Hospital; a DRX-EVOLUTION PIUS (Carestream), the voltages of the lumbar anteroposterior and lateral films were 75-85 kV, and the currents were 15-20 Ams;

Hospital E: YanTai YuHuangDing Hospital; a Digital Diagnost (Philips), the voltages of the lumbar anteroposterior and lateral films were 77-90 kV, and the currents were 33-38 Ams;

3) Detailed information of development processes, parameter, software, packages of the deep learning models

In this study, we determined the most suitable parameters for our PFNet model through the following steps. In addition, as for the model, we examined adjacent context coordination network (ACCoNet)[1], Deepcrack (Deep)[2], and boundary-aware segmentation network (BASNet)[3]. Hyperparameter tuning for the optimizer, learning rate, initial weight, image size, and batch size was performed. For the optimizer, we evaluated the stochastic gradient descent (SGD); The best weight was choose from training epochs (epochs = 200); for the learning rate, the searching range for stochastic gradient descent was 0.0001 and default parameters were used for the other optimizers; for the image size, the searching range was 128, 244, 256, and 512 pixels; for batch size, the searching range was 2–16. For the validation and test set, each image was resized to 512 pixels, and passed through the developed model.

The software liberties and packages we used included python 3.7, opencv-python 4.8.0, pytorch 3.7.1, matplotlib 3.5.3, numpy 1.21.5, pandas 1.3.5, scipy 1.7.3, torchvision 0.10.0, and scikit-learn 1.0.2

Section S2: Supplementary Tables

Supplementary Table 1: Detail baseline characteristics of all external test datasets

	**All external test datasets (n=785)**
**Age (years)**	61.58±14.99
**Sex, no. (%)**	
Male	455 (57.96%)
Female	330 (42.04%)
**Acute VCFs cases, no. (%)**	260 (33.12%)
**Acute VCFs distribution, no. (%)**	
Thoracic	60 (7.64%)
Lumbar	200 (25.48%)

Data are n (%) or mean (SD).

Supplementary Table 2: Detail characteristics of acute VCFs in external test datasets

	All external test datasets (n=785)		
	Acute VCFs (n=260)	non-Acute VCFs (n=525)	P
**Age (years)**	70.11±11.37	57.36±14.78	<0.001
**Sex, no. (%)**			<0.001
Male	72 (27.69%)	258 (49.14%)	
Female	188 (72.31%)	267 (50.86%)	
**Acute VCFs distribution, no. (%)**			
Thoracic	60 (23.08%)	-	
Lumbar	200 (76.92%)	-	

Data are n (%) or mean (SD).

Section S3: Supplementary Figures

Supplementary Figure 1: ROC curves in C-arm fluoroscopy dataset for PFNet model

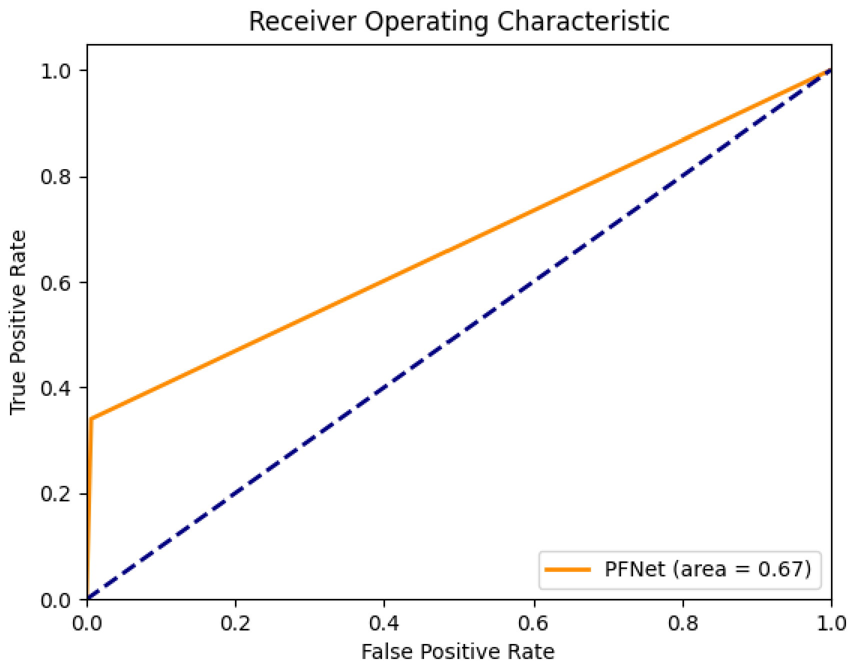

Section S4: References for the Supplementary Material

1. Li G, Liu Z, Zeng D, Lin W, Ling H. Adjacent context coordination network for salient object detection in optical remote sensing images. *IEEE Transactions on Cybernetics.* 2022; 53(1): 526-538.
2. Zou Q, Zhang Z, Li Q, Qi X, Wang Q, Wang S. Deepcrack: Learning hierarchical convolutional features for crack detection. *IEEE transactions on image processing.* 2018; 28(3): 1498-1512.
3. Qin X, Zhang Z, Huang C, Gao C, Dehghan M, Jagersand M. Basnet: Boundary-aware salient object detection. Proceedings of the IEEE/CVF conference on computer vision and pattern recognition. 2019:7479-7489.
